# Dosimetric impact of edema on inguinal lymph node boost in locally advanced vulvar cancer

**DOI:** 10.1002/acm2.13372

**Published:** 2021-08-25

**Authors:** Sandy Mohamed, Lars Fokdal, Marianne S. Assenholt, Jesper Kallehauge, Jacob C. Lindegaard, Kari Tanderup

**Affiliations:** ^1^ Department of Radiation Oncology NCI Cairo University Cairo Egypt; ^2^ Department of Oncology AUH Aarhus Denmark; ^3^ Department of Medical Physics AUH Aarhus Denmark; ^4^ Danish Center for Particle Therapy Aarhus Denmark; ^5^ Institute of Clinical Medicine Aarhus University Aarhus Denmark

**Keywords:** cancer vulva, daily CBCT, groin edema, image guided radiotherapy, inguinal lymph node boost, VMAT

## Abstract

We aimed to evaluate the extent of groin edema and its dosimetric effect in boosted inguinal lymph nodes (LN) for vulvar cancer patients. The level of edema was determined in 10 patients treated with radical radiotherapy. A dosimetric evaluation of six LNs in the patient with the maximum level of edema was performed. The accumulated dose across CBCT fractions was acceptable for all six LNs (>94% of prescribed dose) even with the development of up to 13 mm of edema. The major contributor to fractional dose degradation was geographical displacement of the nodes. We suggest evaluation of edema on daily CBCT.

## 1 INTRODUCTION

Radiotherapy (RT) may be part of the treatment of patients with cancer of the vulva whether definitive, adjuvant, or palliative. Definitive chemoradiotherapy is the treatment of choice for locally advanced vulvar cancer for patients who often need a nodal boost and a high dose of irradiation. Failure after RT for vulvar cancer is most often local.^1^

Lymph node (LN) involvement in vulvar cancer is considered the most important prognostic factor for survival.^2^ Positive LN was statistically correlated with the risk of recurrence with poor prognosis for inguinal and pelvic recurrences.^3^ Hence it is crucial to focus on proper management of positive inguinal LNs and ensure adequate local treatment and dose coverage through the RT course. Furthermore, among all gynecological cancer patients, women treated for vulvar cancer had the highest risk of developing lower limb edema, which can appear subsequent to surgery or RT.^4^

It is technically challenging to deliver IMRT/VMAT to vulva and groins both in terms of patient positioning and dose optimization. There is paucity in the clinical and technical publications focusing on advances in RT management of vulvar cancer and methods for ensuring adequate dose coverage during the RT course. Edema may develop during RT and to our knowledge the dosimetric impact of groin edema on boosted LN has not been quantified and studied, so far. We aimed to measure and evaluate the occurrence of groin edema and its effect on accumulated doses delivered to the boosted inguinal LNs in patients treated with definitive radiochemotherapy for locally advanced vulvar cancer with groin metastases. We aim towards having a more precise RT technique and assuring quality in RT dose delivery. This is what we tried to investigate in our work by evaluating the impact of edema and testing different margins for inguinal LN boosting. We studied in depth the groin edema occurred in six LNs in one patient who had the maximum edema thickness and we expect this to be the worst case among the 10 patients reviewed. We believe if we included patients with less edema, it is not likely to have worse results.

## 2 MATERIAL AND METHODS

Ten consecutive patients with locally advanced vulvar cancer undergoing radical RT with inguinal LN boost were evaluated and the maximum level of groin edema during RT was determined according to the method outlined below.

Patients were PET‐CT and MR scanned in treatment position, and the images were fused according to bony anatomy. Patient fixation was supine with frog leg position. Target volumes included gross tumor volume of the primary (GTV‐T), the pathological lymph nodes (GTV‐N), and the elective vulvar and nodal clinical target volume (CTV‐E). Based on the integrated information from planning PET‐CT (pCT) and MRI individualized internal target volumes (ITV) were defined for CTV‐T, CTV‐N, and CTV‐E. Planning target volume (PTV) was created by adding a 5‐mm margin to the ITV. Each pathologic LN (GTV‐N) was contoured on pCT and MRI.

EBRT was delivered with VMAT: 51.2 Gy/32 fx to the elective target PTV‐E and 64 Gy/32 fx as simultaneously integrated boost to the primary tumor PTV‐T and pathologic LNs PTV‐N. CBCT was acquired at each fraction of RT, rigid bony registration to the pCT was done and daily couch correction was carried out.

**FIGURE 1:**
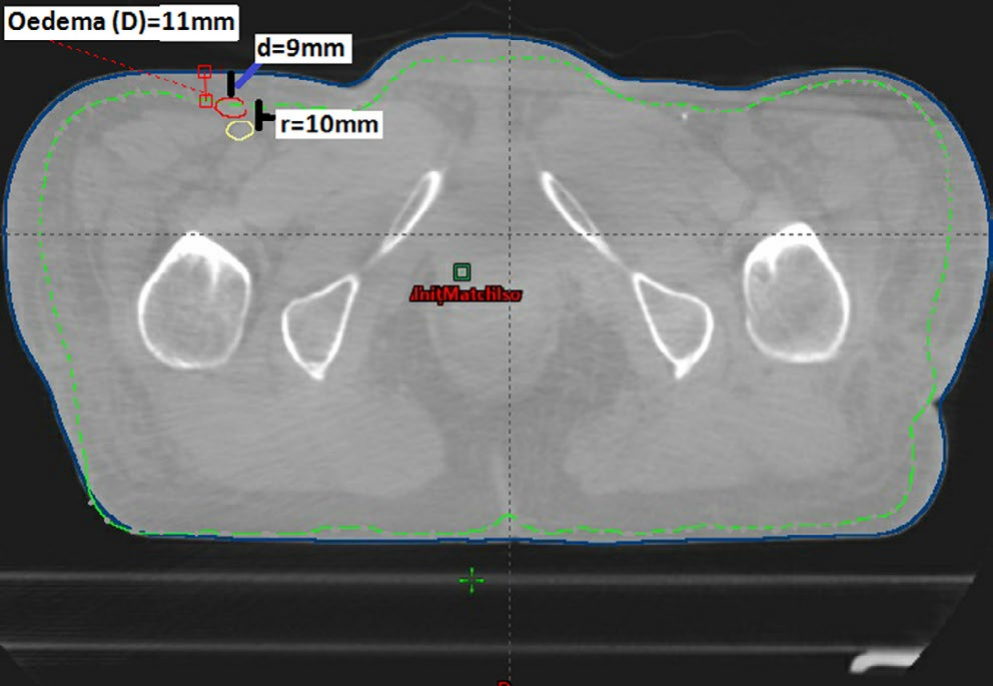
Transverse section of CBCT of fraction number 28 for the presented patient. The outer surface contour is the CBCT contour. The interrupted contour is the planning CT (pCT) body contour. The developed edema is 11 mm on the right inguinal side (difference between pCT and CBCT contours). Red and yellow contours are GTV‐N on CBCT and pCT, respectively. The geographical shift of this LN from pCT to CBCT (center of mass) (“r”) was 10 mm. The depth of this LN from the skin was 9 mm on this CBCT

The body contour was delineated on pCT and on each CBCT. The thickness of the edema during EBRT was defined as the difference between the body contour of pCT and CBCT. The maximum edema was measured in mm at the left and the right groin region on each CBCT at the level of the boosted inguinal LN and named D (Figure 1). The maximum level of edema across all treatment fractions was determined for each patient.

For the patient with the highest measured edema thickness, a dosimetric analysis was carried out based on nine CBCTs evenly distributed across the treatment (including first and last fraction). For each boosted LN the GTV was contoured on the nine CBCTs. The extra volume corresponding to the edema on CBCTs was contoured as a separate volume and included for dose recalculation (as a water equivalent shell) on pCT (Figure 1). The depth from the skin to each LN was measured on each CBCT and named “d” (Figure 1). The geographical shift of the LNs from pCT to CBCT (center of mass) was measured and named “r.”

In order to assess the fractional dose to each LN, dose recalculation was done on pCT taking into account the volume of the edema and the location of the LNs on each CBCT. This was assumed to represent the “delivered dose.” Fractional “delivered” CTV‐Nx D98 was extracted for each LN on each CBCT (in total 6 × 9 = 54 recalculations). “Total delivered” dose was estimated by averaging CTV‐Nx D98 across all nine CBCTs. Planned CTV‐Nx D98 was compared to “total delivered” D98. The comparison was carried out for two plans with 10 mm and 5 mm PTV margin, respectively. These PTV margins were commonly used for inguinal LN boost.

## 3 RESULTS

The maximum level of the measured edema developed during EBRT was median [range] 5 mm [1 mm, 13 mm] across all 10 screened patients.

**FIGURE 2:**
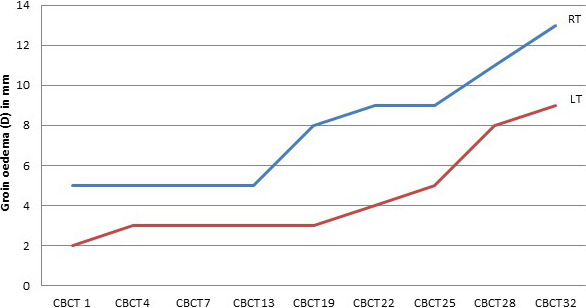
The progression of groin edema (D) measured in mm (*y*‐axis) for the patient who developed maximum level of edema, for right groin represented by the upper line and left groin represented by the lower line. The *x*‐axis shows the number of the corresponding CBCT

The patient with the highest level of edema had six pathologic inguinal LNs (3 left and 3 right). Figure 2 shows the progression of edema during RT for this patient (Figure 2). The median [range] thickness of edema was 5 mm [2–13 mm]. Table 1 shows thickness of edema, LN depth, and CTV D98 for each LN for the 10 mm and 5 mm PTV plans. Total delivered CTV D98 across the six LNs was (median and [range]) 98% [94%, 99%] and 97% [96%–98%] for 10 mm and 5 mm PTV plans, respectively. No statistical difference was found between 10 mm and 5 mm PTV plans (*p* = 0.1). Table 2 gives details about the dose distribution for both plans by showing the conformity, homogeneity indexes, and the coverage of the PTVs for the 5 mm PTV and the 10 mm PTV plans (Table 2).

**TABLE 1:** Planned and delivered dose for the six lymph nodes

	RT1 LN	RT2 LN	RT3 LN	LT1 LN	LT2 LN	LT3 LN
Planning CT: CTV D98% (10 mm PTV) (%)	**98.6**	**97.9**	**99.4**	**94.9**	**99.0**	**99.3**
Planning CT: CTV D98% (5 mm PTV) (%)	**98.8**	**99.0**	**99.8**	**96.2**	**98.8**	**99.6**
**Median** (range) of edema (mm)	**8 [5, 13]**	**8 [5, 13]**	**8 [5, 13]**	**3 [2, 9]**	**3 [2, 9]**	**3 [2, 9]**
LN depth on planning CT (mm)	**5**	**9**	**5**	**1**	**6**	**10**
LN depth on CBCT, **median** (range) in mm	**7 [6, 8]**	**10 [9, 11]**	**7 [5, 13]**	**2 [1, 7]**	**8 [4, 14]**	**13 [11, 20]**
“Total delivered” CTV‐Nx D98 (10 mm PTV) (%)	**96.6**	**99.0**	**98.3**	**94.1**	**98.7**	**98.2**
CTV D98 difference between each CBCT and planning CT (10 mm PTV), **median** (range) (%)	**−0.3 [−9, 0.0]**	**0.4 [−1, 3]**	**−0.4 [−4, −0.1]**	**−1.1 [−6, 2]**	**−0.3 [−3, 1]**	**−0.5 [−3, −0.1]**
“Total delivered” CTV‐Nx D98 (5 mm PTV) (%)	**96.5**	**96.2**	**98.3**	**95.5**	**97.5**	**98.1**
CTV D98 difference between each CBCT and planning CT (5 mm PTV), **median** (range) (%)	**−0.5 [−10.6, 0.2]**	**−0.5[−0.2, −0.8]**	**−0.6 [−5, −0.3]**	**0.01 [−4, 3]**	**−1 [−0.6, −0.1]**	**−0.9 [−4, −0.5]**

**TABLE 2:** The conformity, homogeneity indexes and the coverage of the PTVs for the 5 mm PTV and the 10 mm PTV plans

PTV parameters	5 mm PTV plan	10 mm PTV plan
V95%	99.96	99.87
D98[%]	97.2	96.9
CI(95%)	1.57	1.35
DHI	0.96	0.96
HI	1.09	1.09

Abbreviations: CI, Conformity indexRTOG; DHI, Dose homogeneity index; HI, Homogeneity index.

**FIGURE 3:**
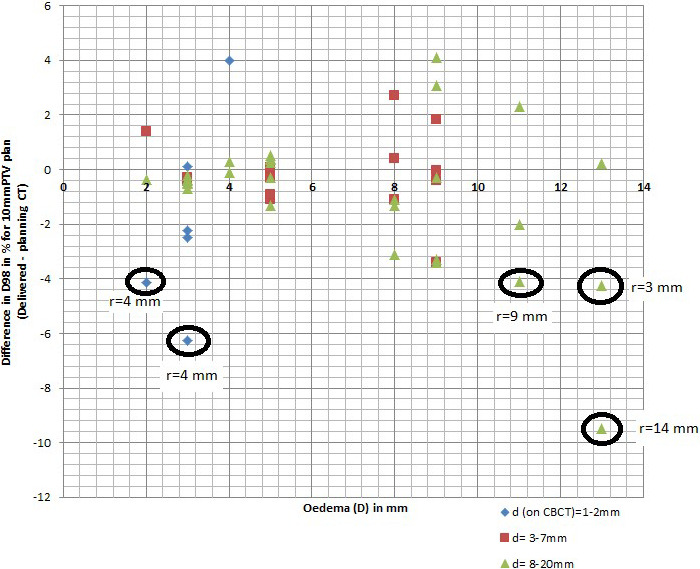
Relation between the edema (D) measured in mm on the *x*‐axis, and difference between the delivered and the pCT CTV D98% in % for the 10 mmPTV plan, on the *y*‐axis. With grouping according to the depth of LN on CBCT measured from skin to the LN (d) in mm where the diamond *d* = 1–2 mm, the square *d* = 3–7 mm, and the triangle *d* = 8–20 mm. *r* = the geographical shift of the LNs from pCT to CBCT (center of mass) is indicated for LNs with dose reduction of more than 4%

**FIGURE 4:**
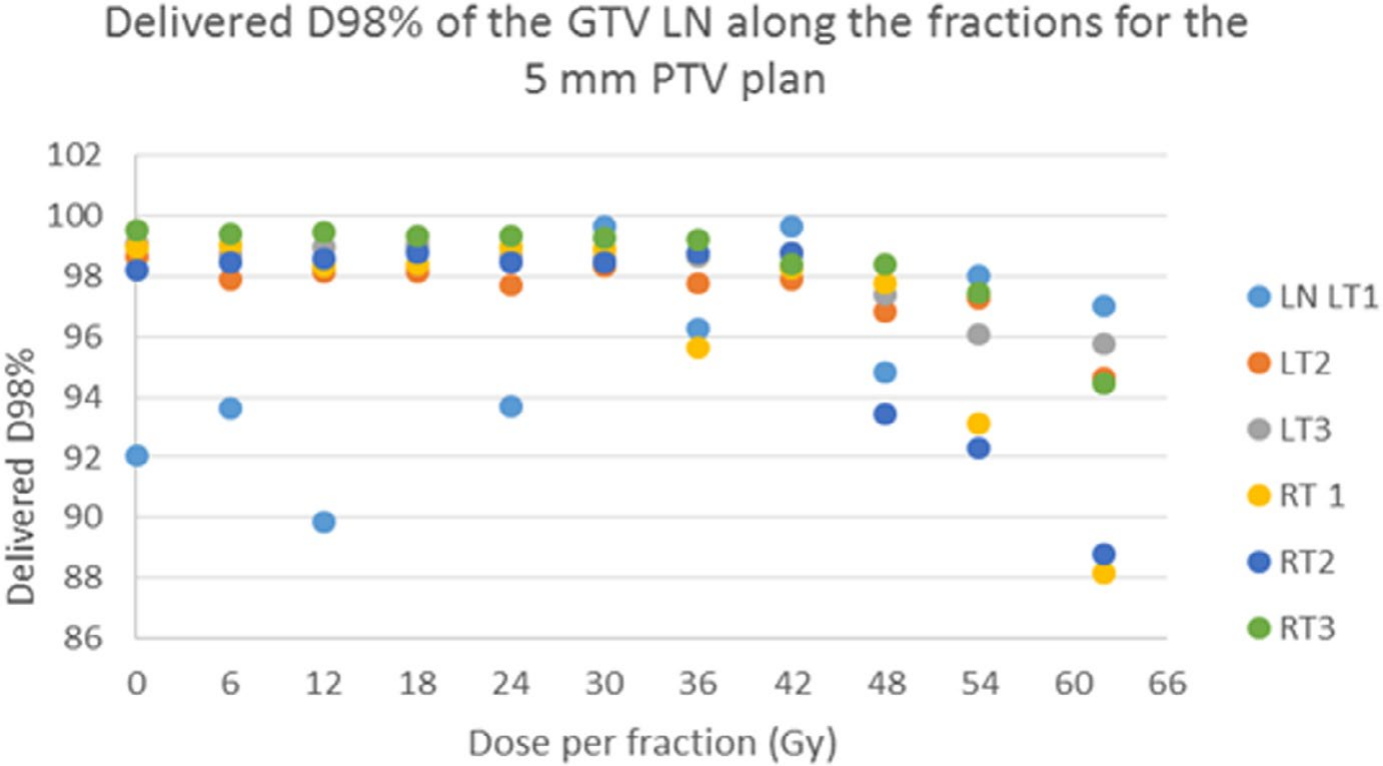
The delivered D98% (y axis) along the course of the treatment (the evaluated CBCTs), for the 5 mm ptv plan for the included six lymph nodes (LN), three on the left inguinal region (LT 1, 2, 3), and three on the right (RT 1,2,3). The x axis shows the dose in Gy of the corresponding CBCT

For LN with geographical shifts <9 mm (PTV 5 mm plans) and <14 mm (PTV 10 mm plans), respectively, the fractional dose difference was <5% for LNs which were located deeper than 2 mm. In LNs with geographical shifts ≥9 mm (5 mm PTV plans) or ≥14 mm (10 mm PTV plans) the dose decreased up to 12% and 9%, respectively, in single fractions. Figure 3 shows for the 10 mmPTV plan the relation between the edema and the difference between the delivered and the pCT CTV D98%. With grouping according to the depth of LN on CBCT measured from skin to the LN. For the 5 mm plan, Figure 4 shows the delivered D98% on each evaluated CBCT this is to show the variation from fraction to fraction.

## 4 DISCUSSION

From our analysis of 10 consecutive vulva cancer patients, we observed groin edema during definitive RT with variable degrees (1–13 mm). We evaluated the dose delivered to pathologic inguinal LN under the condition of development of edema during RT. Regardless of the variable degrees of edema, the accumulated delivered doses with VMAT technique were acceptable for the LNs analyzed. Only VMAT technique dose distribution was assessed here and not 3DCRT or IMRT where fixed beam angles are used.

There are currently no guidelines for actions to be taken towards groin edema, and it is unclear if and when dose recalculation or replanning is needed. Integrated skin flash planning technique has recently been proposed to account for progressive vulvar swelling during RT for vulvar cancer.^5^ The authors concluded that standard IMRT may not sufficiently account for progressive vulvar swelling developed during RT.^5^ However, the authors did not report on the direct dosimetric consequence of edema. Our study did not indicate the need for skin flash for inguinal LNs, which is also typically not possible with VMAT technique.

We measured the edema as a 2D measurement for the depth of edema in relation to the LD. This method is easily implemented and can be carried out directly at the linac. Another method would be to evaluate the edema volume in 3D. However, the volume of edema of interest must be representative to the location of the LN and beam angles should be carefully delineated. It may be interesting to study how 3D identification of the volume of edema performs in relation to the 2D measurement in further studies.

Daily CBCT enables daily patient setup based on bony anatomy. Additionally, CBCT can also be used to detect anatomical changes like edema, LN swelling, and potential geographical shift of the LNs from pCT to CBCT. From our observations, the LN center of mass shift was the major contributor to fractional dose degradations. Furthermore, moderate dose variations (<6%) were seen in LNs situated close to the skin in the build‐up region. We did not observe any linear correlation between the thickness of edema and dose degradation in LNs. This may indicate that the direct effect of photon attenuation caused by the presence of additional tissue was negligible (in this study up to 13 mm). This could be explained by the VMAT dose wash which may justify the minor dosimetric effect of the edema, whereas different results may be obtained with IMRT or 3DCRT where fixed beams are used. However, the thickness of edema can contribute to center of mass shift of LNs and can thereby indirectly cause dose degradation. Therefore, monitoring of edema and LN geographical shift is advisable as early and persistent edema can potentially have clinically relevant dosimetric impact for inguinal LN boosts. Replanning can be done based on the observed LN positions during RT and with adding a water equivalent shell corresponding to the edema.

In our study, no statistical difference was found between 10 mm and 5 mm PTV plans in terms of delivered dose to inguinal LNs. These results may bring to light that a 5 mm margin is adequate. A small margin may make dose escalation possible, as has been demonstrated in cervix cancer radiotherapy.^6^ This has to be tested in a larger cohort of patients. Dose escalation up to 70 Gy to the positive involved inguinal LN was already recommended by different publications.^7^, ^8^

## 5 CONCLUSION

In this study, we found acceptable dosimetric coverage of inguinal LN under the presence of edema up to 13 mm when treated with VMAT. Further studies are needed to substantiate the dosimetric effects of more substantial groin edema. RTT may be trained to measure edema developed during RT and evaluate potential LN displacements. We recommend integrating such procedures in the clinical routine of patient set up.

## AUTHOR CONTRIBUTION

Sandy Mohamed: Corresponding author, contributed with design, execution, data collection, data analysis, interpretation of data and writing of the paper. Lars Fokdal and Jacob Christian Lindegaard: Execution and reviewing. Marianne S. Assenholt: Performing the dose planning. Jesper Kallehague: Data collection and acquisition. Kari Tanderup: Design, execution, data analysis, and interpretation of data for the work, drafting and reviewing. All Authors contributed with editing and approving the submitted manuscript.
